# Bivalent interaction of the PZP domain of BRPF1 with the nucleosome impacts chromatin dynamics and acetylation

**DOI:** 10.1093/nar/gkv1321

**Published:** 2015-11-30

**Authors:** Brianna J. Klein, Uma M. Muthurajan, Marie-Eve Lalonde, Matthew D. Gibson, Forest H. Andrews, Maggie Hepler, Shinichi Machida, Kezhi Yan, Hitoshi Kurumizaka, Michael G. Poirier, Jacques Côté, Karolin Luger, Tatiana G. Kutateladze

**Affiliations:** 1 Department of Pharmacology, University of Colorado School of Medicine, Aurora, CO 80045, USA; 2 Department of Chemistry and Biochemistry and Howard Hughes Medical Institute, University of Colorado, Boulder, CO 80309, USA; 3 St-Patrick Research Group in Basic Oncology, Laval University Cancer Research Center, CHU de Québec Research Center-Oncology Axis, Quebec City, Québec G1R 2J6, Canada; 4 Department of Physics, Ohio State University, Columbus, OH 43210, USA; 5 Graduate School of Advanced Science & Engineering, Waseda University, Tokyo 162-8480, Japan; 6 Goodman Cancer Research Center & Department of Medicine, McGill University, Montreal, Québec H3A 1A1, Canada

## Abstract

BRPF1 (bromodomain PHD finger 1) is a core subunit of the MOZ histone acetyltransferase (HAT) complex, critical for normal developmental programs and implicated in acute leukemias. BRPF1 contains a unique assembly of zinc fingers, termed a PZP domain, the physiological role of which remains unclear. Here, we elucidate the structure-function relationship of this novel epigenetic reader and detail the biological and mechanistic consequences of its interaction with nucleosomes. PZP has a globular architecture and forms a 2:1 stoichiometry complex with the nucleosome, bivalently interacting with histone H3 and DNA. This binding impacts the nucleosome dynamics, shifting the DNA unwrapping/rewrapping equilibrium toward the unwrapped state and increasing DNA accessibility. We demonstrate that the DNA-binding function of the BRPF1 PZP domain is required for the MOZ-BRPF1-ING5-hEaf6 HAT complex to be recruited to chromatin and to acetylate nucleosomal histones. Our findings reveal a novel link between chromatin dynamics and MOZ-mediated acetylation.

## INTRODUCTION

Acetylation of lysine (Kac) is the most frequently occurring post-translational modification (PTM) of histones. This dynamic epigenetic mark is typically present in euchromatin and correlates with high level of gene transcription. The MOZ (monocytic leukemic zinc-finger protein) lysine acetyltransferase 6 (KAT6) is one of the major enzymes that catalyze the acetylation reaction (1–4). Additionally, MOZ associates with and activates a set of DNA-binding transcription factors, including Runx1/2, p53 and PU.1 (5–9). The histone acetyltransferase (HAT) function of the MOZ and homologous MORF complexes regulates expression of *Hox* genes and is essential for normal developmental programs, including formation of brain, heart, blood and bones (5,6,10–17). Aberrant activity of the MOZ and MORF complexes caused by mutations and chromosomal translocations is linked to aggressive forms of leukemia, solid tumors, blood disorders, developmental and cardiac defects, and intellectual disability (4,18–20).

**Figure 1.**
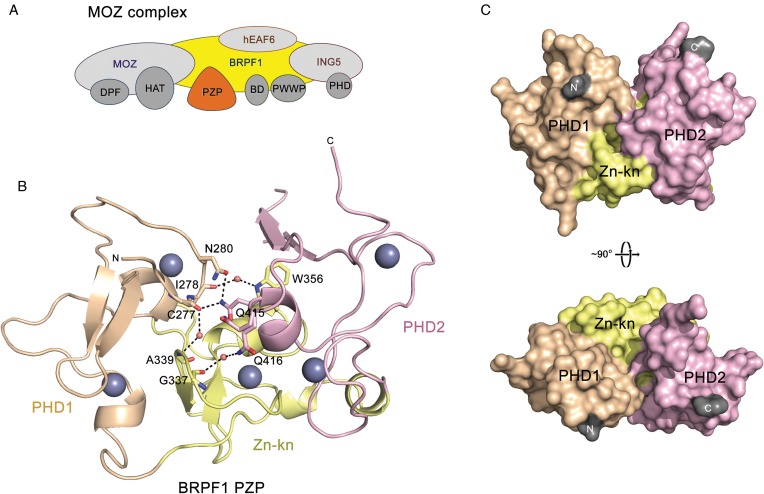
The PZP domain of BRPF1 has a globular architecture. (**A**) The MOZ complex composition. The reader domains in the four core subunits of the complex are labeled. (**B**) The structure of the PZP domain is depicted in a ribbon diagram with PHD1, Zn-kn and PHD2 colored wheat, yellow and pink, respectively. Hydrogen bonds, water molecules and zinc ions are shown as black dashes, salmon spheres and gray spheres, respectively. (**C**) The surface representation of the PZP structure with the N- and C-terminal residues colored gray.

In eukaryotic cells, four core subunits constitute a native MOZ complex (2–4). The catalytic MOZ subunit is coupled to three non-catalytic adaptor proteins: BRPF1 (bromodomain plant homeodomain (PHD) finger 1), ING5 (inhibitor of growth 5) and hEAF6 (homolog of Esa1-associated factor 6) (Figure 1A). Although the precise function of the hEAF6 subunit is unclear, the ING5 subunit recognizes trimethylated Lys4 of histone H3 (H3K4me3) and stabilizes the complex at promoters of actively transcribed genes (21). BRPF1 acts as a scaffolding subunit, linking MOZ, ING5 and hEAF6 together into the functional complex. BRPF1 and its paralogs, BRPF2 and BRPF3, differ in size (1214-, 1058- and 1205-residue, respectively, proteins) but share ∼50% sequence identity and have the same domain architecture.

BRPF1 contains a conserved region that directs specificity of the complex toward a particular histone tail (22) and the MOZ-binding domain (2) in the N-terminus. Three additional domains are present in the C-terminus of the protein, such as a short motif implicated in the interaction with ING5 and hEAF6, acetyllysine-binding bromodomain (BD) and the PWWP domain specific for H3K36me3 (2,23–25). The central region of BRPF1 is characterized by a cysteine-rich sequence encompassing two PHD fingers closely linked by a zinc knuckle (Zn-kn). This conserved arrangement of zinc-binding modules, named a PHD1-Zn-kn-PHD2 or PZP domain, is found in a set of eukaryotic proteins, including Jade1/2/3 (PHF15/16/17) (26,27). Recent studies have shown that the isolated PHD1 finger of BRPF1/2 binds to unmodified histone H3 tail and the second PHD2 finger is capable of associating with DNA (22,28–29). However it remains unclear whether the PZP assembly maintains activities of the individual zinc fingers. Neither the physiological role nor three-dimensional architecture of the intact PZP domain is known.

In this study, we elucidate the structural organization and function of the novel reader domain and detail the biological and mechanistic consequences for binding of the PZP domain to intact nucleosomes. We found that the PZP domain of BRPF1 is a distinct functional unit that forms a 2:1 stoichiometry complex with nucleosome, bivalently interacting with both the histone H3 tail and DNA and preferring a nucleosome with extra-nucleosomal linker DNA. Multivalent binding of the PZP domain to the nucleosome affects the nucleosome dynamics. It stabilizes the particle in a more open state, facilitating binding of a transcription factor to its target DNA sequence, which is less accessible in the fully wrapped nucleosome. We demonstrate that the DNA-binding function of the BRPF1 PZP domain is required for the MOZ-BRPF1-ING5-hEaf6 HAT complex to associate with chromatin and acetylate histones.

## MATERIALS AND METHODS

### DNA cloning and protein purification

The BRPF1 PZP (amino acids 267–454), PHD1 (amino acids 267–334) and PHD2 (amino acids 374–454) domains, cloned into a pDEST15 vector, were expressed in *Escherichia coli* Rosetta-2 (DE3) pLysS cells grown in either Luria Broth or minimal media supplemented with ^15^NH_4_Cl (Sigma) and 150 μM ZnCl_2_. After induction with 0.5–1.0 mM IPTG for 16 h at 16°C, bacteria were harvested through centrifugation and lysed by sonication. GST fusion proteins were purified on glutathione Agarose 4B beads (Fisher) and the GST tag was either cleaved with PreScission protease, or left on for electrophoretic mobility shift assay (EMSA) experiments. The GST-tagged proteins were eluted off the beads with reduced L-glutathione (Fisher). The proteins were further purified using Q Sepharose and size exclusion (SEC) chromatography and concentrated in Millipore concentrators (Millipore).

### PCR mutagenesis

The triple mutants of the BRPF1 PZP domain, C277S/C284S/C361S (PZP_3x_), K383E/K390E/R392E and K422E/R427E/R439E, were generated using the Stratagene QuickChange XL Site Directed Mutagenesis protocol. All mutant sequences were confirmed by DNA sequencing.

### X-ray crystallography

The PZP domain of BRPF1 (amino acids 267–454) was concentrated to ∼4 mg/ml in 50 mM Tris–HCl pH 7.5, supplemented with 100 mM KCl and 1 mM tris(2-carboxyethyl)phosphine (TCEP). The protein solution was incubated overnight with the unmodified H3 (residues 1–12) peptide in a 1:2 molar ratio prior to crystallization. Crystals were grown using the sitting-drop diffusion method at 25°C by mixing 600 nl of protein-peptide solution with 600 nl of well solution composed of 0.2 M Calcium acetate, 0.1 M Imidazole pH 8 and PEG8000 10% (w/v). Although the histone H3 peptide was present, the PZP domain was crystallized in an apo-state. X-ray diffraction data were collected from a single crystal at 100 K on a ‘NOIR-1’ detector system at the Molecular Biology Consortium Beamline 4.2.2 of the Advance Light Source (ALS). The Asn280-Asn289 and Met423-Arg439 regions of PZP are highly dynamic as reflected by high B-factor values and poor electron density. Protein structure was solved using Single-wavelength Anomalous Dispersion method with Zn anomalous signal. Datasets were processed with XDS, and location of the Zn^2+^ atoms were found with Phenix AutoSol (30). The starting model was generated with Phenix Autobuild, and refinement was carried out with Phenix.refine. Manual model building was performed in Coot (31) and structure was verified by MOLProbity (32). The crystallographic statistics are shown in Supplementary Table S1.

### Nucleosome assembly

Mononucleosomes, nucleosome core particles (NCP)165 and NCP147, were assembled using 165 bp or 147 bp DNA containing the ‘601’ positioning sequence (33). The 147 bp DNA construct was assembled into NCP with no DNA overhangs, while the 165 bp DNA had 7 and 11 extra-nucleosomal bp linker DNA on either side. Nucleosomes were assembled either with unlabeled *Xenopus* octamer or Atto647N labeled *Xenopus* octamer labeled at H4E63C as described (34).

### Analytical ultracentrifugation

Unlabeled NCP165 was incubated with increasing molar ratios of PZP overnight at 4°C in a buffer containing 20 mM Tris 7.5, 50 mM NaCl, and 2 mM arginine. The samples were then diluted to 400 μl with the same buffer and centrifuged at 20°C using a Beckman XLI An50Ti rotor and a speed of 40 000 rpm until the entire sample was sedimented. Data was analyzed and diffusion coefficients for S value corrections were estimated using Ultrascan III and LIMS (Demeler, http:/www.ultrascan.uthscsa.edu), as described (35).

### Protein labeling

Purified PZP was labeled at its native cysteine residues with Oregon Green 488 fluorophore as described (36). Excess fluorophore was removed by washing with buffer containing 25 mM HEPES pH 8.0, 0.1 mM ethylenediaminetetraacetic acid (EDTA), 500 mM NaCl and 0.1 mM TCEP. A typical labeling efficiency of 40–60% was routinely obtained.

### HIFI-FRET assay

**Figure 2.**
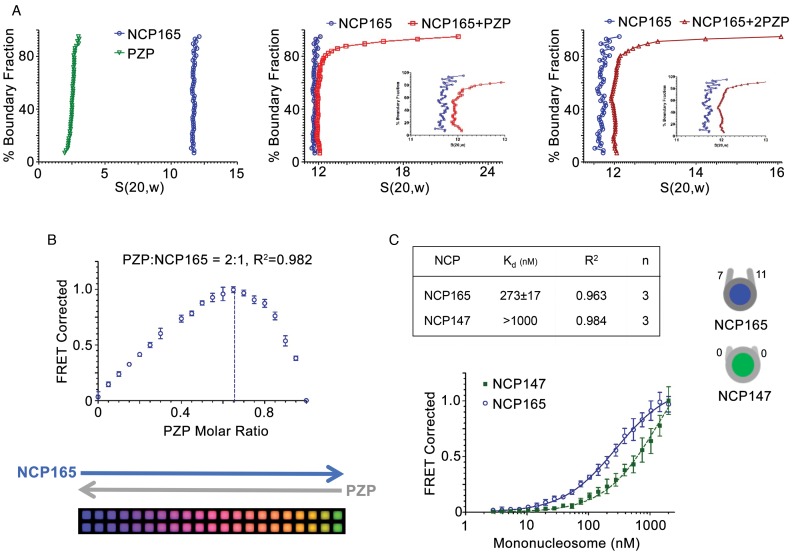
The PZP domain binds mononucleosomes. (**A**) Sedimentation velocity profiles for NCP165 and PZP in the free state (left), for the 1:1 PZP–NCP165 complex (middle) and for 2:1 PZP-NCP165 complex (right). (**B**) Job's plot for the NCP165–PZP complex formation. The molar ratio of PZP was estimated to be 0.65 and that of NCP165 was estimated to be 0.35, indicated by the peak of the curve (dashed line). These values yielded the stoichiometric ratio of ∼2:1 (PZP:NCP165). (**C**) FRET-derived binding curves for the interaction of PZP with NCP165 and NCP147. The binding curve for NCP147 did not plateau and an apparent *K*_d_ with a lower limit of 1000 nM (*R*^2^ = 0.984) was determined. Each experiment was performed in triplicate with different mononucleosome preparations. Error bars are the SD of three individual experiments.

Atto647N-labeled mononucleosomes were titrated against three different concentrations of Oregon Green labeled PZP, in a buffer containing 25 mM Tris 7.5, 200 mM NaCl, 0.01% (v/v) each of NP40 and CHAPS. FRET corrections and calculations were performed as described (37) with a modification: the data were fit in GraphPad Prism using a quadratic equation. Each binding experiment contained replicates and each experiment was performed three times with different nucleosome preparations. Error reported in Figure 2C is the standard deviation of each experiment. To determine stoichiometry, we used Job plot assay, in which the total molar concentration of reactants was kept constant, 1 μM. The FRET corrected values were plotted in Graphpad prism and fit with cubic polynomial equation. The assay and analysis was performed essentially as described (36).

### Cy3-Cy5 nucleosome and LexA preparation for FRET measurements

Nucleosomal DNA, 601L was prepared by PCR from a plasmid containing the LexA binding site at bases 8–27 with the Cy3-labeled oligonucleotide, Cy3-CTGGAGATACTGTATGAGCATACAGTACAATTGGTC and the unlabeled oligonucleotide, ACAGGATGTATATATCTGACACGTGCCTGGAGACTA. The Cy3 labeled oligonucleotide was labeled using a Cy3-NHS esther (GE Healthcare) at a 5’ amino group and purified by RP-HPLC on a 218TPTM C18 (Grace/Vydac) column. Human histones, including H3C110A, and LexA were expressed and purified by known methods (38,39). Labeling of recombinant H2AK119C and H2B with Cy5-maleamide and reconstitution of Cy3-Cy5 labeled NCP was performed as described (40).

FRET efficiency measurement experiments were carried out on a Horiba Scientific Fluoromax 4. Samples were excited at 510 and 610 nm and the photoluminescence spectra were measured from 530 to 750 nm and 630 to 750 nm for donor and acceptor excitations, respectively. Each wavelength was integrated for 1 s, and the excitation and emission slit width were set to 5 nm with 2 nm emission wavelength steps. FRET measurements were computed through the (ratio)_A_ method.

**Figure 3.**
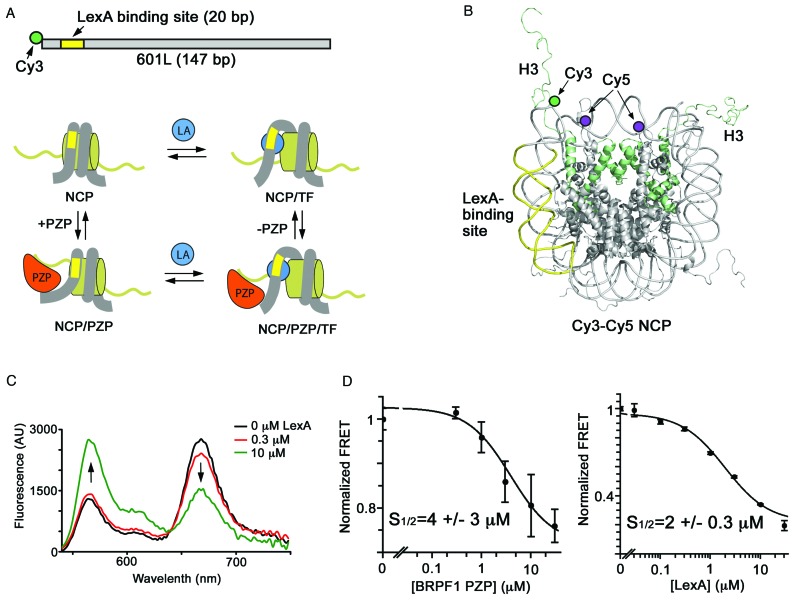
FRET measurements of the effect of binding of BRPF1 PZP to NCP on the NCP dynamics. (**A**) Diagram of the 601L DNA construct and a model of LexA (LA) binding to its target site within the nucleosome. (**B**) Crystal structure of the NCP (PDB ID: 1KX5) that indicates the positions of H3 (green ribbon), Cy5 at H2AK119C (purple circle), Cy3 at the 5 prime end of 601L (green circle) and the LexA target site (yellow). (**C**) Fluorescence spectra recorded on Cy3-Cy5 labeled NCP in the presence of 0–10 μM of LexA. The Cy3 emission increases as the Cy5 emission decreases indicating a decrease in FRET efficiency. (**D**) FRET efficiencies of BRPF1 PZP and LexA titrations. Error bars represent a standard deviation based on 3 experiments. The BRPF1 PZP titration included 2 μM of LexA.

LexA titrations were carried out in either 19 mM Tris pH 7.8, 75 mM NaCl, 0.006% Tween20 and 30 μM EDTA or 12 mM Tris pH 7.8, 75 mM NaCl, 5% glycerol, 5 mM DTT, 0.005% Tween20 with 10 nM nucleosomes. PZP titrations were carried out with a LexA equal to the measured S_1/2_ of LexA binding to NCPs (1–2 μM). FRET values in each titration were normalized to the FRET efficiency in the absence of the titrant. Titrations were fit to *E* = (*E*_f_ – *E*_0_)/(1 + (S_1/2_/C) + *E*_0_, where E is the FRET efficiency at concentration C of the titrant, *E*_0_ the efficiency in the absence of the titrant, *E*_f_ the efficiency at high titrant concentration, and S_1/2_ is the inflection point. Errors in Figure 3 and Supplementary Figure S2 represent a standard deviation based on three experiments.

### NMR experiments

Nuclear magnetic resonance (NMR) experiments were performed at 298K on a Varian INOVA 600 MHz spectrometer using pulse field gradients to suppress artifacts and eliminate water signal. ^1^H,^15^N heteronuclear single quantum coherence (HSQC) spectra of uniformly ^15^N-labeled BRPF1 PZP, PZP_3x_ or PHD2 (0.1–0.2 mM) in 25 mM Tris–HCl pH 7.0 or 7.5 buffer, 150 mM NaCl, 5 mM dithiothreitol and ∼8% D_2_O were collected as histone H3 peptides (aa 1–12 and aa 1–19) (synthesized by the University of Colorado Denver Peptide Core Facility) or and/or 601 Widom DNA or 14 bp DNA were added stepwise into the protein samples. NMR data were processed and analyzed with NMRPipe and NMRDraw as described (41). The 14 bp DNA (5’-CCTACAGCAAAGC) oligos (IDT) were annealed by combining in equal molar amounts, heating to 95°C and slowly cooled to room temperature.

### Electrophoretic mobility shift assay

A total of 32 repeats of the 601 Widom DNA sequence were cloned into the pJ201 plasmid and transformed into DH5α cells. The plasmid was purified as described (40) and by Qiagen-QIAprep Spin Miniprep kit. Separation of the individual sequences was completed by digestion of the plasmid with EcoRV. The 601 Widom DNA was purified from the remaining plasmid by gel extraction (Qiagen-MinElute Gel Extraction kit). For EMSA experiments NCPs were reconstituted using recombinant human core histones as described (42).

Increasing amounts of GST-PZP_3x_ were incubated with 601 Widom DNA (3 pmol/lane) or with NCP (3 pmol/lane) in buffer (25 mM Tris-HCl pH 7.5, 150 mM NaCl, 5 mM dithiothreitol with or without 10% glycerol) for 30 minutes at room temperature. The following concentrations of GST-PZP_3x_ were used in experiments shown in Figure 5A: 0, 50, 100, 200, 460 μM (as well as 460 μM GST-PZP_3x_ only, left gel). The following concentrations of each GST-PZP, wild-type (WT) and K383E/K390E/R392E and K422E/R427E/R439E mutants, were used in the experiment shown in Figure 5E (with 601 DNA (2 pmol/lane)): 8, 16, 33 and 45 μM. The reaction mixtures were loaded on 5% native polyacrylamide gels and electrophoresis was performed in 0.2× TB buffer (1× TB = 90 mM Tris, 64.6 mM boric acid) at 125–150 V on ice. Gels were stained with ethidium bromide and visualized by AlphaImager (AlphaInnotech).

### Purification of WT and mutant MOZ-BRPF1-ING5-hEaf6 complexes and HAT assays

Purification of protein complexes after co-transfection was performed as previously described (26,27). Briefly, 293T cells were transfected with each subunit (HA-BRPF1 (wt or mutants), Flag-MOZ, Flag- or HA-ING5 and Flag- or HA-hEaf6) and harvested 48 h later. Flag or HA IP using anti-Flag M2 agarose beads (Sigma) or anti-HA agarose beads (Roche) was performed followed by elution with 3× Flag or HA peptides. HAT assays were performed as described previously (22). Briefly, native human chromatin and free histones were used to carry out HAT assays in a 15 μl reaction containing 50 mM Tris–HCl pH 8.0, 10% glycerol, 1 mM DTT, 0.1 mM EDTA, 1 mM PMSF, 10 mM sodium butyrate (Sigma) and 1.25 nCi ^3^H labeled acetyl-CoA (Perkin Elmer Life Sciences). Samples were spotted on P81 membranes (GE Healthcare) for scintillation counting.

## RESULTS

### PZP has a globular architecture

To characterize the PZP assembly of BRPF1, we performed ^1^H,^15^N HSQC experiments on the uniformly ^15^N-labeled PZP domain and individual PHD1 and PHD2 fingers and overlaid the spectra (Supplementary Figure S1; we were unable to generate a soluble construct of Zn-kn). Little to no overlap in resonances of the isolated PHD fingers and PZP indicated a distinctively different chemical environment for the PHD fingers when they are assembled into the PZP domain. To define the spatial organization of PZP, we obtained a 2.05 Å-resolution crystal structure of the BRPF1 region, encompassing PHD1, Zn-kn and PHD2 (residues 267–454). The structure shows a globular architecture of PZP with the three domains being fully integrated (Figure 1). Five zinc-binding clusters formed by the C4HC5HC5HC2H topological motif stabilize the overall fold. The Zn-kn acts as a platform orienting PHD1 and PHD2 in a way that the N- and C-termini of PZP are faced the same direction (Figure 1B and C). The three domains are joined through a zipper-like network of hydrogen bonds and hydrophobic interactions (Figure 1B). The structure of PZP reveals a unique fold for zinc-coordinating modules and suggests that this domain may represent a distinct functional unit.

### BRPF1 PZP forms a complex with mononucleosome

To assess if the PZP domain associates with chromatin *in vitro*, we reconstituted NCPs containing a 165 bp DNA fragment (NCP165) and examined binding of the BRPF1 PZP domain to NCP165 in analytical ultracentrifuge (AUC) sedimentation velocity (SV) experiments (Figure 2A). The diffusion-corrected sedimentation coefficient distributions (S) of the PZP domain and NCP165 alone indicated that both components are present in a monomeric and homogenous state in solution (Figure 2A, left panel). An addition of the PZP domain to solution of NCP165 resulted in faster sedimentation of NCP165, implying that a higher molecular weight complex (NCP165-PZP) was formed (Figure 2A, middle panel). Unexpectedly, when the PZP concentration was increased beyond the 1:1 ratio, assuming that one molecule of PZP binds to one NCP165, S_avg_ value further increased. Addition of two PZP molecules per nucleosome resulted in an S_avg_ of 12.1, indicating the formation of a higher order complex.

To determine the precise stoichiometry of the PZP–nucleosome complex, we analyzed a Job plot in the continuous variation method paired with HIFI-FRET. We labeled the BRPF1 PZP domain with Oregon Green 488 donor fluorophore at its native cysteine residues, whereas NCP165 was labeled with Atto647N acceptor fluorophore at histone H4E63C. The fluorescently labeled PZP and NCP165 samples were incubated in a manner that a total concentration of the reactants was maintained constant at 1 μM, with the concentration of PZP being progressively increased from 0 nM and the concentration of NCP165 being progressively decreased from 1 μM in 50 nM titration steps (Figure 2B). Analysis of the FRET signal in the resultant Job plot yielded a peak value at 0.65, which corresponds to 0.65 molar ratio for PZP and 0.35 molar ratio for NCP165. These values translate to a stoichiometry of 1.8:1 for the PZP:NCP165 complex, supporting the AUC-SV results that two BRPF1 PZP molecules can associate with one nucleosome.

We next measured the dissociation constant (*K*_d_) for the nucleosome–PZP complex by HI-FI FRET (Figure 2C). An apparent *K*_d_ of ∼273 nM was determined by titrating Atto647N-labeled NCP165 against the Oregon Green 488-labeled PZP. In contrast, the interaction of PZP with a nucleosome assembled on a shorter, 147 bp DNA sequence (NCP147), was weaker, >1 μM. Together, these data suggest that an extra-nucleosomal linker DNA is essential for tight binding of the BRPF1 PZP domain to nucleosomes.

### Binding of BRPF1 PZP to nucleosome stabilizes an open form of NCP

We subsequently examined the impact of binding of the BRPF1 PZP domain to NCP on the equilibrium between nucleosome unwrapping and rewrapping. We prepared NCP containing the 147 bp 601 nucleosome positioning sequence (601L) with the transcription factor LexA binding site replacing bases 8–27 (Figure 3A and B). The Cy3 donor fluorophore was attached to the 5′ end of the 601L DNA adjacent to the LexA site, and the Cy5 acceptor fluorophore was attached to histone H2A(K119C) (40). This placed Cy3 in the proximity of one of the Cy5 fluorophores, therefore a significant FRET signal is expected from a fully wrapped nucleosome, while a reduced FRET is expected when the NCP is in a more open, partially unwrapped state (43,44). Titration of LexA into Cy3-Cy5 labeled NCP caused a decrease in FRET due to LexA binding to its target site and stabilization of the unwrapped state (40,43–44). Consistent with previous measurements, the value for the LexA concentration at which FRET efficiency is reduced by 50% (S_1/2_) was found to be 2 ± 0.3 μM (Figure 3C and D).

To investigate the effect of BRPF1 PZP on the nucleosome unwrapping/rewrapping equilibrium, we first titrated BRPF1 PZP with Cy3-Cy5 labeled nucleosomes and found that BRPF1 PZP does not induce a measurable change in the FRET efficiency over the range at which it binds histone tails (data not shown). This suggests that BRPF1 PZP binding itself does not shift the equilibrium. However when BRPF1 PZP was titrated into NCP with LexA at a fixed concentration equal to the LexA S_1/2_, the FRET efficiency was reduced substantially, resulting in a S_1/2_ = 4 μM for BRPF1 PZP (Figure 3D and Supplementary Figure S2). These results indicate that binding of the PZP domain to NCP shifts the unwrapping/rewrapping equilibrium toward the unwrapped state, enhancing the DNA accessibility within the nucleosome to other DNA-binding proteins, transcription factors and co-activators.

### The histone H3 binding mechanism is conserved in BRPF1 PZP

**Figure 4.**
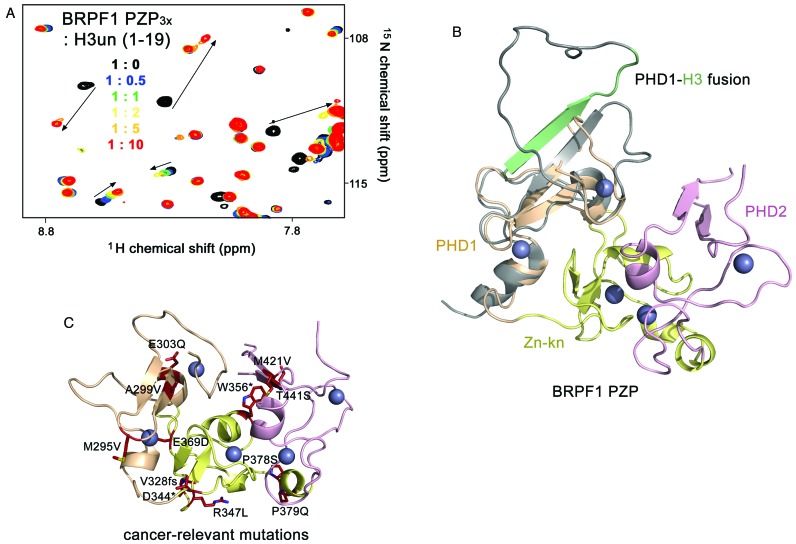
BRPF1 PZP interacts with the unmodified histone H3 tail. (**A**) Overlay of ^1^H, ^15^N HSQC spectra of BRPF1 PZP_3x_ in the presence of increasing concentrations of unmodified H3 peptide (residues 1–19 of H3). The spectra are color-coded according to the protein-peptide ratio. (**B**) Superimposed structures of the BRPF1 PZP domain and the PHD1 finger of orthologous BRPF2 fused to a sequence corresponding to histone H3 tail (PDB ID: 2L43). The BRPF1 PZP structure is colored as in Figure 1. The BRPF2 PHD1 finger with a long artificial linker is gray, and the fused unmodified histone H3 tail is green. (**C**) Residues of the BRPF1 PZP domain, found mutated in cancer, are shown as sticks in red and labeled.

The BRPF1 PZP domain contains 20 cysteine residues, 16 of which coordinate five zinc ions and four are free cysteines that likely account for a significant fraction, up to 90% of aggregated protein when it is expressed in *E. coli*. To increase the yield of properly folded PZP, we mutated three free cysteine residues and tested the triple C277S/C284S/C361S (PZP_3x_) mutant in NMR experiments. As the PHD1 finger in BRPF1 and BRPF2 has previously been shown to bind to the first seven residues of unmodified histone H3 tail (22,28), we titrated unlabeled H3 peptide (residues 1–19 of H3) into the ^15^N-labeled PZP_3x_ NMR sample (Figure 4A). Substantial chemical shift perturbations (CSPs) were observed in ^1^H,^15^N HSQC spectra of the PZP_3x_ domain, corroborating binding. Furthermore, the pattern of CSPs induced in PZP_3x_ was comparable to the pattern of CSPs induced in the WT PZP by the unmodified H3 peptide, confirming that PZP_3x_ and PZP bind to the H3 tail equally well (*K*_d_∼12 μM) (22).

We superimposed the structures of the BRPF1 PZP domain and the orthologous BRPF2 PHD1 finger fused to a sequence corresponding to the histone H3 tail (Figure 4B) (28). The superimposition shows that the histone-binding site of the PHD1 finger is located on the side, which is opposite to the interface of PHD1 with Zn-kn-PHD2. The histone-binding site is not occluded in the BRPF1 PZP domain, pointing to a likely similar mechanism for recognition of histone H3 tail. To explore the contribution of Zn-kn and PHD2 in the context of the intact PZP domain, we carried out NMR titrations with H3K9ac and H3K14ac peptides (residues 1–19 of H3) (Supplementary Figure S3). The BRPF1 PZP_3x_ domain recognized H3K9ac and H3K14ac peptides essentially to the same extent as it recognized unmodified H3. These results suggest that Zn-kn or PHD2 are not involved in the interaction with residues 1–19 of H3, in contrast to the double PHD finger (DPF) of the catalytic MOZ subunit, where binding to histone H3 is enhanced through acetylation and both PHD fingers are engaged in the interaction with H3K14ac (45–47).

### BRPF1 PZP bivalently binds to histone H3 and DNA

**Figure 5.**
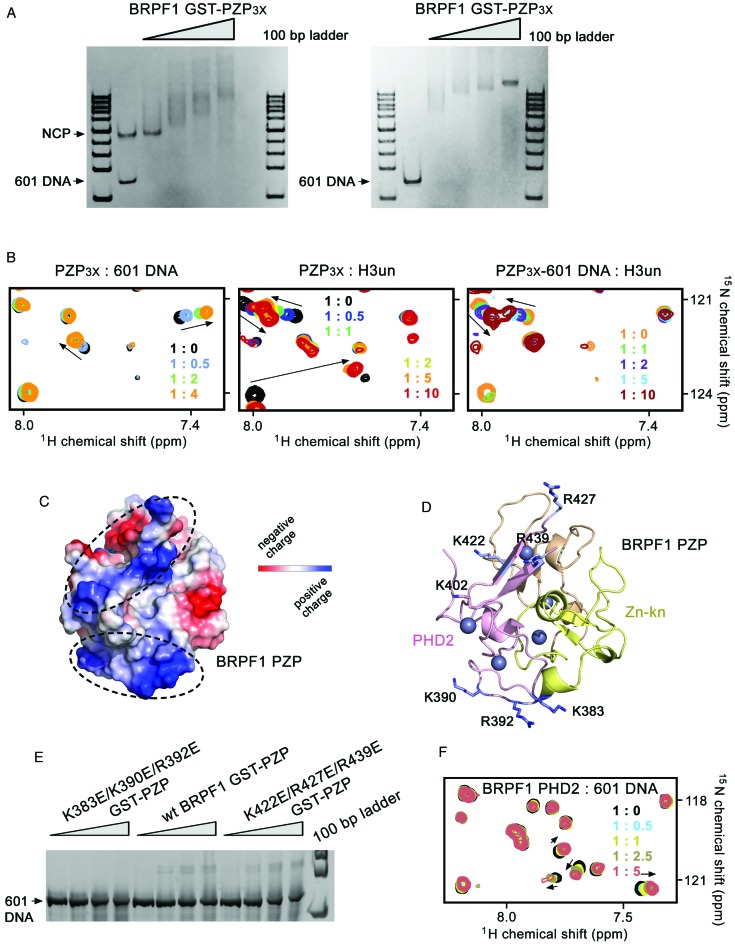
BRPF1 PZP bivalently binds to histone H3 and DNA. (**A**) EMSA with NCP147 (left) or 147 bp 601 DNA only (right) incubated with increasing amounts of GST-PZP_3x_, as described in ‘Materials and Methods’ section. The experiments were repeated at least three times. (**B**) Overlays of ^1^H, ^15^N HSQC spectra of BRPF1 PZP_3x_ in the presence of increasing concentrations of 601 DNA (left) or unmodified H3 peptide (middle). The right panel shows an overlay of ^1^H, ^15^N HSQC spectra of the 601 DNA-bound PZP_3x_ in the presence of increasing amounts of unmodified H3 peptide. The spectra are color-coded according to the protein-ligand ratio (inset). (**C**) Electrostatic potential surface representation of the BRPF1 PZP domain generated in PyMol with basic clusters outlined by dashed circles. (**D**) A ribbon diagram of the PZP domain shown in the same orientation as in (C). The positively charged residues in the basic clusters are labeled. (**E**) EMSA with 601 DNA in the presence of increasing amounts of the indicated GST-PZP proteins, either wt or K383E/K390E/R392E and K422E/R427E/R439E mutants, as described in ‘Materials and Methods’ section. (**F**) Superimposed ^1^H, ^15^N HSQC spectra of the BRPF1 PHD2 finger, collected as 601 DNA was titrated in.

Approximately 45-fold tighter binding of the BRPF1 PZP domain to nucleosome as compared to its binding to histone H3 tail suggested additional contacts. To examine whether the BRPF1 PZP domain associates with DNA, we employed EMSA. NCP, reconstituted with a 147 bp 601 Widom DNA sequence or 147 bp 601 DNA alone were incubated with increasing amounts of GST-PZP_3x_ and the reaction mixtures were resolved on a 5% native polyacrylamide gel (Figure 5A and Supplementary Figure S4A). A gradual increase in amount of added GST-PZP_3x_ resulted in a shift of the free DNA and NCP bands, indicating formation of the GST-PZP_3x_/DNA and GST-PZP_3x_/NCP, respectively, complexes. We note that the free 601 DNA band shifted faster than the NCP band, implying that the PZP domain prefers a more accessible, free DNA to the DNA wrapped around the nucleosome, which is in agreement with FRET analysis shown in Figure 2C.

We investigated binding of the BRPF1 PZP domain to DNA in more detail by titrating 601 DNA to the ^15^N-labeled PZP_3x_ sample while collecting ^1^H,^15^N HSQC spectra of the protein (Figure 5B, left panel). Large CSPs caused by 601 DNA confirmed the interaction. Analysis of CSPs induced in the PZP_3x_ domain by 601 DNA and separately by histone H3 peptide revealed that each ligand perturbs an entirely different set of amides (compare left and middle panels in Figure 5B). A lack of mutually perturbed residues suggested that the binding sites for H3 and DNA in the PZP domain do not overlap. This conclusion was further supported by NMR experiments in which the histone H3 peptide was titrated into the PZP_3x_–DNA complex and, *vice* *versa*, 601 DNA was titrated into the PZP_3x_–H3 complex (Figure 5B, right panel and data not shown). Either ligand induced a reproducible pattern of CSPs in a set of amides regardless of the presence or absence of another ligand. These data demonstrate that the PZP_3x_ domain of BRPF1 is capable of binding both DNA and H3 simultaneously.

To identify the DNA-binding site in the BRPF1 PZP domain, we analyzed its electrostatic surface potential (Figure 5C). The presence of apparent basic clusters on the protein surface suggested that they could be involved in binding to the negatively charged DNA. The Zn-kn-PHD2 region is particularly enriched in Arg and Lys residues, having a PI value of 8.3, as compared to the PI value of 4.1 for the PHD1 finger. One of the basic clusters contains a Zn-kn residue K383 and the K390 and R392 residues of PHD2, whereas the second cluster contains K422, R427 and R439 of PHD2 (Figure 5D). We generated the triple mutants of PZP, K383E/K390E/R392E and K422E/R427E/R439E, and tested their interactions with 601 DNA by EMSA. As shown in Figure 5E, the K383E/K390E/R392E mutant of PZP was incapable of binding to DNA, whereas the K422E/R427E/R439E mutant retained the DNA binding activity. These data suggest that the K383, K390 and R392 residues of the Zn-kn and PHD2 finger are required for strong interaction with DNA. Interestingly, the isolated PHD2 finger of BRPF1 also bound to 601 DNA, however much weaker and non-specifically based on similar CSPs observed in the PHD2 finger upon binding to a 14 bp DNA (Figure 5F and Supplementary Figure S4b). In contrast, the 14 bp DNA was unable to induce CSPs in the PZP_3x_ domain, most likely due to the fact that some of the surface Arg and Lys residues in the isolated PHD2 finger are buried within the PZP domain (Supplementary Figure S4c).

### DNA binding of BRPF1 is required for HAT activity of the MOZ complex but is dispensable for the MOZ complex assembly

**Figure 6.**
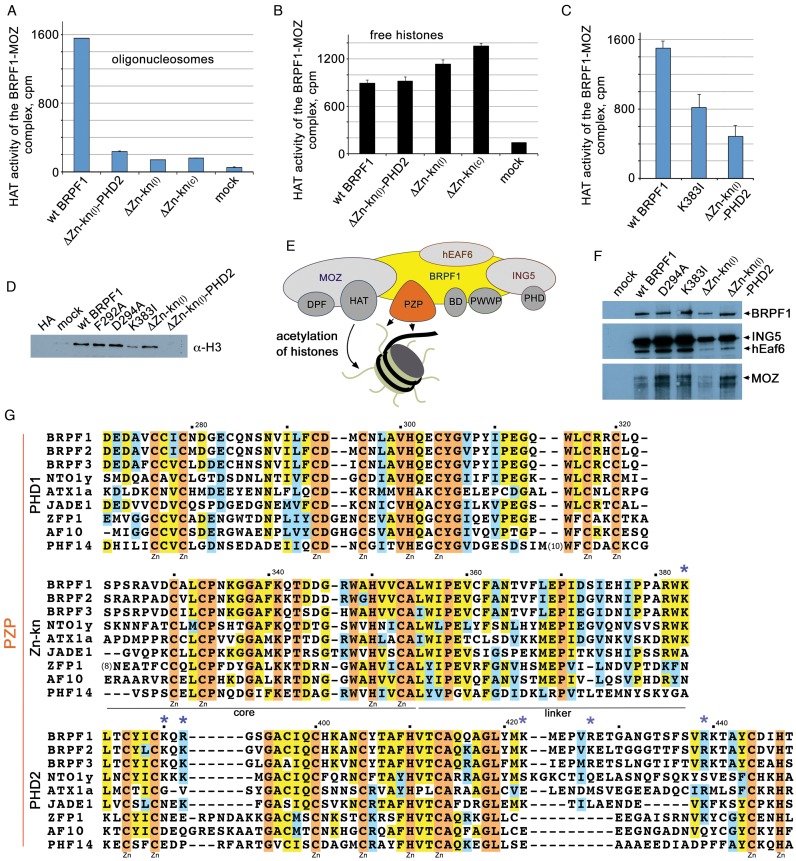
DNA binding of BRPF1 is required for HAT activity of the MOZ complex but is dispensable for the MOZ complex assembly. (**A**) PHD2 and Zn-kn of BRPF1 subunit are required for acetylation of chromatin. HAT assays on oligonucleosomes using wild-type (WT) or mutant BRPF1 complexes (MOZ-BRPF1-ING5-hEaf6) from 293T cells. The core region and the linker region of Zn-kn are abbreviated as (C) and (l), respectively. ΔZn-kn_(c)_ (Δ aa 334–356), ΔZn-kn_(l)_ (Δ aa 359–384) and ΔZn-kn_(l)_-PHD2 (Δ aa 359–450). (**B**) Deletions in the BRPF1 PZP domain do not abrogate acetylation of free histones. HAT assays on free histones using WT or mutant BRPF1 complexes from 293T cells. Error bars in (A–**C**) are the SD based on triplicate (C) and duplicate (A and B) assays. (C) The K383I point mutation in Zn-kn decreases chromatin acetylation by the MOZ-BRPF1-ING5-hEaf6 HAT complex *in vitro*. HAT assays on oligonucleosomes using WT or mutated complexes from 293T cells. (**D**) HAT complexes that harbour PHD2 or Zn-kn mutations in the BRPF1 subunit are defective in pulling down endogenous chromatin when purified from 293T cells. The complexes were loaded on SDS-PAGE gel. Western blot analysis using anti-H3 antibodies was performed to assess chromatin-binding activity. (**E**) Schematic representation of the MOZ-BRPF1-ING5-hEaf6 complex and its activity on chromatin. Interaction of BRPF1 PZP with nucleosomes promotes acetylation of histones by the HAT domain of MOZ. (**F**) Deletions and mutations in the PZP domain do not affect the MOZ-BRPF1-ING5-hEaf6 complex assembly. Western blot analysis using anti-Flag antibody for detection of the Flag-MOZ subunit and anti-HA antibody for detection of HA-BRPF1 WT and mutants, HA-hEaf6 and HA-ING5 subunits shows co-purification of the four subunits. (**G**) Alignment of the PZP sequences: absolutely, moderately and weakly conserved residues are colored orange, yellow and blue, respectively. Zinc-coordinating residues are specified. Each tenth residue of BRPF1 is marked by a dot and labeled. Asterisks indicate basic residues mutated in this study.

To assess the role of the BRPF1 PZP–DNA interaction in acetylation function of the MOZ-BRPF1-ING5-hEaf6 complex, we immunopurified the complex from co-transfected HEK293 cells and tested its enzymatic activity on human oligonucleosomes and free histones (Figure 6A–C and Supplementary Figure S5). The purified complex containing WT BRPF1 acetylated oligonucleosomes with high efficiency, whereas free histones were acetylated to a lesser degree. We generated mutant constructs of full length BRPF1 carrying deletions in Zn-kn and PHD2, including ΔZn-kn_(c)_ (Δ aa 334–356), ΔZn-kn_(l)_ (Δ aa 359–384) and ΔZn-kn_(l)_-PHD2 (Δ aa 359–450) and a point mutation K383I. We then transfected cells with the mutant BRPF1 constructs, purified the corresponding MOZ-BRPF1-ING5-hEaf6 complexes, and tested them in HAT assays. We found that the HAT activity of all mutant complexes was either substantially compromised or completely abrogated on oligonucleosomes. Importantly, this was not seen using free histones as substrates, indicating that the DNA binding by the Zn-kn-PHD2 portion of the BRPF1 PZP domain is required for the MOZ complex to acetylate chromatin.

To test whether the compromised HAT activity is due to the impaired ability of the mutant BRPF1 proteins to associate with chromatin, we analyzed the purified MOZ-BRPF1-ING5-hEaf6 complexes by western blot (Figure 6D and F). As expected, the WT MOZ-BRPF1-ING5-hEaf6 complex was capable of pulling down endogenous chromatin (Figure 6D). Substitution of the histone-binding site residues in the PHD1 finger, F292 and D294, with an alanine did not affect the recruitment of the mutant complexes to chromatin, whereas chromatin binding ability of the MOZ complexes harboring K383I, ΔZn-kn_(l)_ or ΔZn-kn_(l)_-PHD2 alterations in BRPF1 was notably decreased. All the BRPF1 mutants tested were capable of forming the tetrameric complexes (Figure 6F). Together, these data demonstrate that the DNA binding function of the BRPF1 PZP domain is necessary for the MOZ complex to associate with and acetylate chromatin and that this domain is not involved in the MOZ complex assembly.

## DISCUSSION

In this work we characterize the structure–function relationship of the novel reader domain and detail the biological and mechanistic consequences for its binding to intact nucleosomes. We found that the PZP domain of BRPF1 is a distinct functional unit with a unique globular organization. The three fused zinc-binding fingers bind tightly to NCPs through interacting with both the histone H3 tail and DNA, preferring an extra-nucleosomal or linker DNA. Two BRPF1 PZP molecules can form complexes with a single nucleosome, engaging two histone H3 tails and two DNA (one entry and one exit) sites. Interaction of BRPF1 PZP with NCPs facilitates binding of a transcription factor to its target DNA sequence, which is less accessible in the wrapped NCP. This suggests a shift in equilibrium toward a more open form of the nucleosome and hindering the rewrapping event, most likely due to decreasing *k*_on_. This mechanism for facilitating nucleosome unwrapping and enhancing DNA accessibility could be essential for binding to DNA by a set of transcription factors and co-activators known to interact with and be activated by MOZ, such as p53, Runx1/2 and PU.1 (5–9). Our findings reveal that the DNA-binding function of the BRPF1 PZP domain is required for the MOZ-BRPF1-ING5-hEaf6 HAT complex to be recruited to chromatin and to acetylate histones, and that PZP is not involved in the MOZ-BRPF1-ING5-hEaf6 complex assembly (Figure 6E).

Of the four subunits of the MOZ-BRPF1-ING5-hEaf6 complex, three harbor PTM readers, such as BD, the PWWP domain and a number of zinc fingers, whose organization and functions differ substantially (Figures 1A and 6E). The tandem PHD fingers in the catalytic MOZ subunit are coupled to form a distinct module, the double PHD finger (DPF). The BRPF1 subunit contains the PZP assembly, and a typical single PHD finger is seen in ING5. Why does the MOZ complex need so many PTM readers? Clearly, the coordinated binding of multiple readers should enhance apparent affinity and specificity of the complex. Such combinatorial readout of the epigenetic environment can provide a lock and key–type mechanism to recruit or stabilize the MOZ complex at a particular genomic site, which in turn is a prerequisite to elicit a desired biological outcome (3,48). Further studies are needed to better understand crosstalk between the PTM readers in the MOZ-BRPF1-ING5-hEaf6 complex and the significance of this crosstalk for normal signaling processes and oncogenesis.

Aberrant HAT activity of the MOZ-BRPF1-ING5-hEaf6 complex is associated with a number of human cancers. In most cases, misregulation of the complex is caused by chromosomal translocations and mutations in the subunits. According to the cBioPortal for Cancer Genomics site, BRPF1 is found amplified in 27% of breast cancers or mutated in 9% of stomach cancers. To date, 159 cancer-relevant mutations, including reading frame-shift and translational termination aberrations, have been identified in BRPF1, with 12 of them in the PZP domain (Figure 4C). Our structural study demonstrates that many of these residues are found at the zinc-finger interfaces, suggesting that the structural stability of the PZP domain may be compromised in the cancer-relevant mutants.

In addition to BRPF1/2/3, the PZP module is found in a range of proteins that also form translocation chimeras in acute leukemias, including JADE1/2/3 and AF10/17. An alignment of amino acid sequences of a set of the PZP domain-containing proteins reveals that the histone binding residues in the PHD1 finger are relatively conserved (Figure 6G). However many basic residues in Zn-kn and PHD2 are absent in the ATX1a, JADE1, AF10 and PHF14 proteins, implying that, in contrast to BRPF1, these may not bind DNA. It will be essential to explore this possibility and compare activities of the PZP domains, as our knowledge regarding functions of this set of epigenetic readers remains very limited.

## ACCESSION NUMBER

Atomic coordinates for the structure of the BRPF1 PZP domain have been deposited in Protein Data Bank under accession code 5ERC.

## SUPPLEMENTARY DATA

Supplementary Data are available at NAR Online.

SUPPLEMENTARY DATA

## References

[1] Doyon Y., Cayrou C., Ullah M., Landry A.J., Cote V., Selleck W., Lane W.S., Tan S., Yang X.J., Cote J. (2006). ING tumor suppressor proteins are critical regulators of chromatin acetylation required for genome expression and perpetuation. Molecular cell.

[2] Ullah M., Pelletier N., Xiao L., Zhao S.P., Wang K., Degerny C., Tahmasebi S., Cayrou C., Doyon Y., Goh S.L. (2008). Molecular architecture of quartet MOZ/MORF histone acetyltransferase complexes. Molecular and cellular biology.

[3] Klein B.J., Lalonde M.E., Cote J., Yang X.J., Kutateladze T.G. (2014). Crosstalk between epigenetic readers regulates the MOZ/MORF HAT complexes. Epigenetics.

[4] Yang X.J. (2015). MOZ and MORF acetyltransferases: Molecular interaction, animal development and human disease. Biochimica et biophysica acta.

[5] Perez-Campo F.M., Borrow J., Kouskoff V., Lacaud G. (2009). The histone acetyl transferase activity of monocytic leukemia zinc finger is critical for the proliferation of hematopoietic precursors. Blood.

[6] Katsumoto T., Aikawa Y., Iwama A., Ueda S., Ichikawa H., Ochiya T., Kitabayashi I. (2006). MOZ is essential for maintenance of hematopoietic stem cells. Genes Dev..

[7] Rokudai S., Aikawa Y., Tagata Y., Tsuchida N., Taya Y., Kitabayashi I. (2009). Monocytic leukemia zinc finger (MOZ) interacts with p53 to induce p21 expression and cell-cycle arrest. J. Biol. Chem..

[8] Pelletier N., Champagne N., Stifani S., Yang X.J. (2002). MOZ and MORF histone acetyltransferases interact with the Runt-domain transcription factor Runx2. Oncogene.

[9] Collins H.M., Kindle K.B., Matsuda S., Ryan C., Troke P.J., Kalkhoven E., Heery D.M. (2006). MOZ-TIF2 alters cofactor recruitment and histone modification at the RARbeta2 promoter: differential effects of MOZ fusion proteins on CBP- and MOZ-dependent activators. J. Biol. Chem..

[10] Sheikh B.N., Downer N.L., Phipson B., Vanyai H.K., Kueh A.J., McCarthy D.J., Smyth G.K., Thomas T., Voss A.K. (2015). MOZ and BMI1 play opposing roles during Hox gene activation in ES cells and in body segment identity specification in vivo. Proceedings of the National Academy of Sciences of the United States of America.

[11] Vanyai H.K., Thomas T., Voss A.K. (2015). Mesodermal expression of Moz is necessary for cardiac septum development. Developmental biology.

[12] Sheikh B.N., Lee S.C., El-Saafin F., Vanyai H.K., Hu Y., Pang S.H., Grabow S., Strasser A., Nutt S.L., Alexander W.S. (2015). MOZ regulates B-cell progenitors and, consequently, Moz haploinsufficiency dramatically retards MYC-induced lymphoma development. Blood.

[13] You L., Zou J., Zhao H., Bertos N.R., Park M., Wang E., Yang X.J. (2015). Deficiency of the chromatin regulator BRPF1 causes abnormal brain development. J. Biol. Chem..

[14] Voss A.K., Collin C., Dixon M.P., Thomas T. (2009). Moz and retinoic acid coordinately regulate H3K9 acetylation, Hox gene expression, and segment identity. Dev. Cell.

[15] Thomas T., Corcoran L.M., Gugasyan R., Dixon M.P., Brodnicki T., Nutt S.L., Metcalf D., Voss A.K. (2006). Monocytic leukemia zinc finger protein is essential for the development of long-term reconstituting hematopoietic stem cells. Genes Dev..

[16] Crump J.G., Swartz M.E., Eberhart J.K., Kimmel C.B. (2006). Moz-dependent Hox expression controls segment-specific fate maps of skeletal precursors in the face. Development.

[17] You L., Yan K., Zhou J., Zhao H., Bertos N.R., Park M., Wang E., Yang X.J. (2015). The lysine acetyltransferase activator Brpf1 governs dentate gyrus development through neural stem cells and progenitors. PLoS Genet..

[18] Arboleda V.A., Lee H., Dorrani N., Zadeh N., Willis M., Macmurdo C.F., Manning M.A., Kwan A., Hudgins L., Barthelemy F. (2015). De novo nonsense mutations in KAT6A, a lysine acetyl-transferase gene, cause a syndrome including microcephaly and global developmental delay. Am. J. Hum. Genet..

[19] Tham E., Lindstrand A., Santani A., Malmgren H., Nesbitt A., Dubbs H.A., Zackai E.H., Parker M.J., Millan F., Rosenbaum K. (2015). Dominant mutations in KAT6A cause intellectual disability with recognizable syndromic features. Am. J. Hum. Genet..

[20] Sheikh B.N., Phipson B., El-Saafin F., Vanyai H.K., Downer N.L., Bird M.J., Kueh A.J., May R.E., Smyth G.K., Voss A.K. (2015). MOZ (MYST3, KAT6A) inhibits senescence via the INK4A-ARF pathway. Oncogene.

[21] Champagne K.S., Saksouk N., Pena P.V., Johnson K., Ullah M., Yang X.J., Cote J., Kutateladze T.G. (2008). The crystal structure of the ING5 PHD finger in complex with an H3K4me3 histone peptide. Proteins.

[22] Lalonde M.E., Avvakumov N., Glass K.C., Joncas F.H., Saksouk N., Holliday M., Paquet E., Yan K., Tong Q., Klein B.J. (2013). Exchange of associated factors directs a switch in HBO1 acetyltransferase histone tail specificity. Genes Dev..

[23] Laue K., Daujat S., Crump J.G., Plaster N., Roehl H.H., Kimmel C.B., Schneider R., Hammerschmidt M. (2008). The multidomain protein Brpf1 binds histones and is required for Hox gene expression and segmental identity. Development.

[24] Vezzoli A., Bonadies N., Allen M.D., Freund S.M., Santiveri C.M., Kvinlaug B.T., Huntly B.J., Gottgens B., Bycroft M. (2010). Molecular basis of histone H3K36me3 recognition by the PWWP domain of Brpf1. Nat. Struct. Mol. Biol..

[25] Poplawski A., Hu K., Lee W., Natesan S., Peng D., Carlson S., Shi X., Balaz S., Markley J.L., Glass K.C. (2013). Molecular Insights into the Recognition of N-Terminal Histone Modifications by the BRPF1 Bromodomain. J. Mol. Biol..

[26] Avvakumov N., Lalonde M.E., Saksouk N., Paquet E., Glass K.C., Landry A.J., Doyon Y., Cayrou C., Robitaille G.A., Richard D.E. (2012). Conserved molecular interactions within the HBO1 acetyltransferase complexes regulate cell proliferation. Mol. Cell. Biol..

[27] Saksouk N., Avvakumov N., Champagne K.S., Hung T., Doyon Y., Cayrou C., Paquet E., Ullah M., Landry A.J., Cote V. (2009). HBO1 HAT complexes target chromatin throughout gene coding regions via multiple PHD finger interactions with histone H3 tail. Mol. Cell.

[28] Qin S., Jin L., Zhang J., Liu L., Ji P., Wu M., Wu J., Shi Y. (2011). Recognition of unmodified histone H3 by the first PHD finger of bromodomain-PHD finger protein 2 provides insights into the regulation of histone acetyltransferases monocytic leukemic zinc-finger protein (MOZ) and MOZ-related factor (MORF). J. Biol. Chem..

[29] Liu L., Qin S., Zhang J., Ji P., Shi Y., Wu J. (2012). Solution structure of an atypical PHD finger in BRPF2 and its interaction with DNA. J. Struct. Biol..

[30] Adams P.D., Afonine P.V., Bunkoczi G., Chen V.B., Davis I.W., Echols N., Headd J.J., Hung L.W., Kapral G.J., Grosse-Kunstleve R.W. (2010). PHENIX: a comprehensive Python-based system for macromolecular structure solution. Acta Crystallogr. D Biol. Crystallogr..

[31] Emsley P., Lohkamp B., Scott W.G., Cowtan K. (2010). Features and development of Coot. Acta crystallogr. D Biol. Crystallogr..

[32] Chen V.B., Arendall W.B., Headd J.J., Keedy D.A., Immormino R.M., Kapral G.J., Murray L.W., Richardson J.S., Richardson D.C. (2010). MolProbity: all-atom structure validation for macromolecular crystallography. Acta Crystallogr. D Biol. Crystallogr..

[33] Lowary P.T., Widom J. (1998). New DNA sequence rules for high affinity binding to histone octamer and sequence-directed nucleosome positioning. J. Mol. Biol..

[34] Clark N.J., Kramer M., Muthurajan U.M., Luger K. (2012). Alternative modes of binding of poly(ADP-ribose) polymerase 1 to free DNA and nucleosomes. J. Biol. Chem..

[35] Rogge R.A., Kalashnikova A.A., Muthurajan U.M., Porter-Goff M.E., Luger K., Hansen J.C. (2013). Assembly of nucleosomal arrays from recombinant core histones and nucleosome positioning DNA. J. Vis. Exp..

[36] Muthurajan U.M., Hepler M.R., Hieb A.R., Clark N.J., Kramer M., Yao T., Luger K. (2014). Automodification switches PARP-1 function from chromatin architectural protein to histone chaperone. Proc. Natl. Acad. Sci. U.S.A..

[37] Hieb A.R., D'Arcy S., Kramer M.A., White A.E., Luger K. (2012). Fluorescence strategies for high-throughput quantification of protein interactions. Nucleic Acids Res..

[38] Luger K., Rechsteiner T.J., Richmond T.J. (1999). Expression and purification of recombinant histones and nucleosome reconstitution. Methods Mol. Biol..

[39] Little J.W., Kim B., Roland K.L., Smith M.H., Lin L.L., Slilaty S.N. (1994). Cleavage of LexA repressor. Methods Enzymol..

[40] Musselman C.A., Gibson M.D., Hartwick E.W., North J.A., Gatchalian J., Poirier M.G., Kutateladze T.G. (2013). Binding of PHF1 Tudor to H3K36me3 enhances nucleosome accessibility. Nat. Commun..

[41] Klein B.J., Piao L., Xi Y., Rincon-Arano H., Rothbart S.B., Peng D., Wen H., Larson C., Zhang X., Zheng X. (2014). The histone-H3K4-specific demethylase KDM5B binds to its substrate and product through distinct PHD fingers. Cell Rep..

[42] Tachiwana H., Kagawa W., Osakabe A., Kawaguchi K., Shiga T., Hayashi-Takanaka Y., Kimura H., Kurumizaka H. (2010). Structural basis of instability of the nucleosome containing a testis-specific histone variant, human H3T. Proc. Natl. Acad. Sci. U.S.A..

[43] Li G., Widom J. (2004). Nucleosomes facilitate their own invasion. Nat. Struct. Mol Biol..

[44] North J.A., Amunugama R., Klajner M., Bruns A.N., Poirier M.G., Fishel R. (2013). ATP-dependent nucleosome unwrapping catalyzed by human RAD51. Nucleic Acids Res..

[45] Dreveny I., Deeves S.E., Fulton J., Yue B., Messmer M., Bhattacharya A., Collins H.M., Heery D.M. (2014). The double PHD finger domain of MOZ/MYST3 induces alpha-helical structure of the histone H3 tail to facilitate acetylation and methylation sampling and modification. Nucleic Acids Res..

[46] Qiu Y., Liu L., Zhao C., Han C., Li F., Zhang J., Wang Y., Li G., Mei Y., Wu M. (2012). Combinatorial readout of unmodified H3R2 and acetylated H3K14 by the tandem PHD finger of MOZ reveals a regulatory mechanism for HOXA9 transcription. Genes Dev..

[47] Ali M., Yan K., Lalonde M.E., Degerny C., Rothbart S.B., Strahl B.D., Cote J., Yang X.J., Kutateladze T.G. (2012). Tandem PHD fingers of MORF/MOZ acetyltransferases display selectivity for acetylated histone H3 and are required for the association with chromatin. J. Mol. Biol..

[48] Musselman C.A., Lalonde M.E., Cote J., Kutateladze T.G. (2012). Perceiving the epigenetic landscape through histone readers. Nat. Struct. Mol. Biol..

