# Supplementary material for: Bivalent interaction of the PZP domain of BRPF1 with the nucleosome impacts chromatin dynamics and acetylation
Source: Nucleic Acids Res. 2015 Nov 30;44(1):472–84. doi: 10.1093/nar/gkv1321 (PMC4705663; doi:10.1093/nar/gkv1321)
Supplement: SUPPLEMENTARY DATA [file supp_44_1_472__index.html]

Bivalent interaction of the PZP domain of BRPF1 with the nucleosome impacts chromatin dynamics and acetylation — Bivalent interaction of the PZP domain of BRPF1 with the nucleosome impacts chromatin dynamics and acetylation — SUPPLEMENTARY DATA 

# Bivalent interaction of the PZP domain of BRPF1 with the nucleosome impacts chromatin dynamics and acetylation

## SUPPLEMENTARY DATA

- SUPPLEMENTARY DATA
