# Supplementary material for: Bivalent interaction of the PZP domain of BRPF1 with the nucleosome impacts chromatin dynamics and acetylation
Source: Nucleic Acids Res. 2015 Nov 30;44(1):472–84. doi: 10.1093/nar/gkv1321 (PMC4705663; doi:10.1093/nar/gkv1321)
Supplement: SUPPLEMENTARY DATA [file supp_gkv1321_nar-03034-h-2015-File008.pdf]

Supplementary Information

Bivalent interaction of the PZP domain of BRPF1 with the nucleosome impacts chromatin dynamics and acetylation

Brianna J. Klein[1#], Uma M. Muthurajan[2#], Marie-Eve Lalonde[3], Matthew D. Gibson[4], Forest H. Andrews[1], Maggie Hepler[2], Shinichi Machida[5], Kezhi Yan[6], Hitoshi Kurumizaka[5], Michael G. Poirier[4], Jacques Côté[3], Karolin Luger[2*] and Tatiana G. Kutateladze[1*]

[1]Department of Pharmacology, University of Colorado School of Medicine, Aurora, CO 80045, USA

[2]Department of Chemistry and Biochemistry and Howard Hughes Medical Institute, University of Colorado, Boulder, CO 80309, USA

[3]St-Patrick Research Group in Basic Oncology, Laval University Cancer Research Center, CHU de Québec Research Center-Oncology Axis, Quebec City, Québec G1R 2J6, Canada

[4]Department of Physics, Ohio State University, Columbus, Ohio 43210, USA

[5]Graduate School of Advanced Science & Engineering, Waseda University, Tokyo 162-8480, Japan

[6]McGill Cancer Center & Department of Medicine, McGill University, Montreal, Québec H3A 1A1, Canada

[#]Equal contribution

[*]Correspondence to: Tatiana G. Kutateladze, tatiana.kutateladze@ucdenver.edu or Karolin Luger Karolin.Luger@Colorado.edu

Supplementary Figure S1. Superimposed $^{1}H,^{15}N$ HSQC spectra of PZP_{3x}, PZP, and the individual PHD1 and PHD2 fingers.

Supplementary Figure S2. FRET efficiencies of BRPF1 PZP titrations (a) without and (b) with 1 μM LexA measured in buffer containing 5% glycerol and 5 mM DTT. Error bars represent a standard deviation based on 3 experiments.

Supplementary Figure S3. Superimposed $^{1}H,^{15}N$ HSQC spectra of BRPF1 PZP_{3x} , recorded while the H3K9ac and H3K14ac peptides (residues 1-19 of H3) were titrated in.

Supplementary Figure S4. (a) EMSA with 601 DNA (3 pmol/lane) in the presence of increasing amounts of wt GST-PZP. Concentrations of wt GST-PZP are as follows: 0 μM, 5 μM, 10 μM, 100 μM, 150 μM, 200 μM, 300 μM, as well as 300 μM with no 601 DNA and a DNA ladder. (b, c) Spectral overlays for $^{1}H,^{15}N$ HSQC titrations of BRPF1 PHD2 (b) or PZP_{3x} (c) in the presence of increasing concentrations of 14 mer annealed dsDNA.

Supplementary Figure S5. HAT assays were performed on free histones using purified wt or mutant MOZ-BRPF1-ING5-hEaf6 complexes. 293T cells were transfected with Flag-MOZ, Flag-ING5, Flag-hEaf6 and wt or mutant HA-BRPF1 subunits. Flag IP was used for complex purification followed by elution with 3xFlag peptide. Error bars are the SD based on triplicate assays.

Klein et al., Suppl. Fig. S1

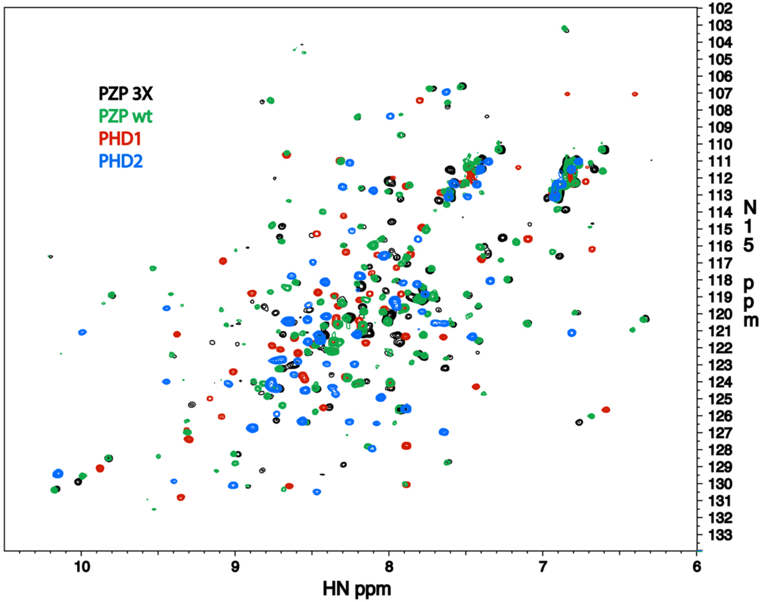

Klein et al., Suppl. Fig. S2

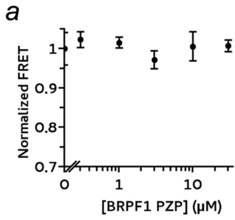

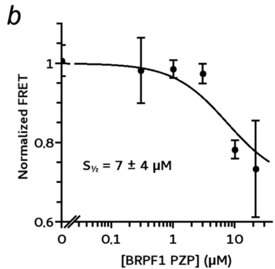

Klein et al., Suppl. Fig. 3S

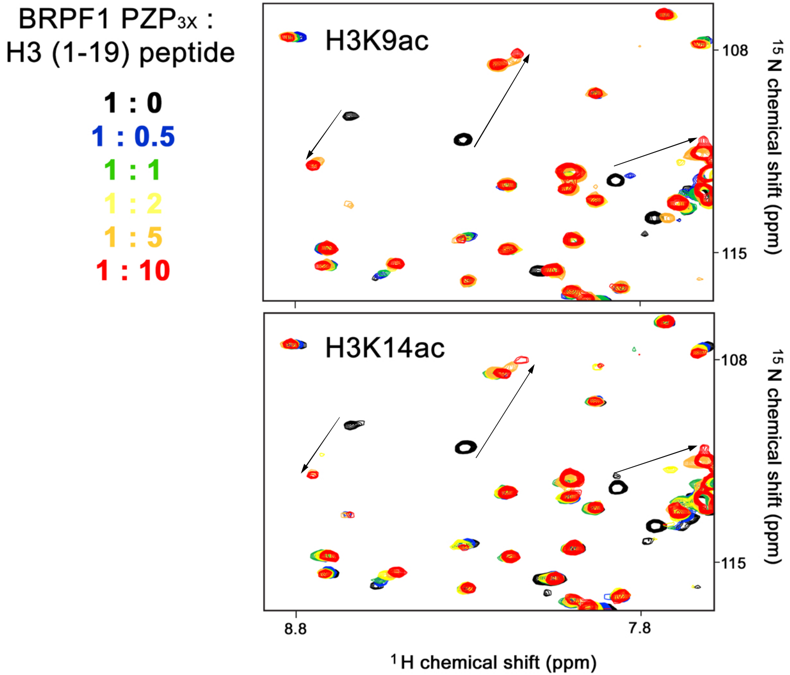

Klein et al., Suppl. Fig. S4

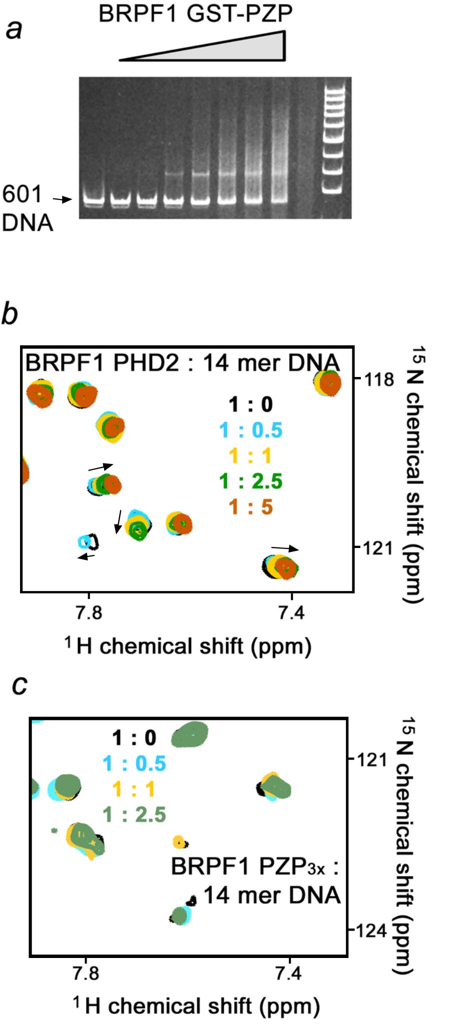

Klein et al., Suppl. Fig. S5

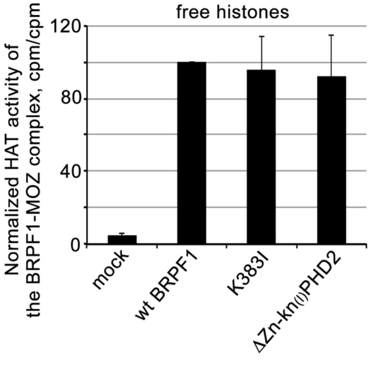

Supplementary Table 1. Crystal structure data collection and refinement statistics

	PZP
**Data collection**	
Space group	P_{6122}
Cell dimensions	
a, *b*, *c* (Å)	73.87, 73.87, 147.13
a, *b*, *g* (°)	90, 90, 120
Resolution (Å)	2.05 (2.12-2.05)*
R_{sym} or R_{merge}	16.7 (66.4)
Mean $I/\sigma I$	19.6 (4.6)
Completeness (%)	100 (100)
Redundancy	21.5 (17)
**Refinement**	
Resolution (Å)	2.05-63.97
No. reflections	15595
$R_{work/}$ R_{free}	16.22/19.34
No. atoms	1464
Protein/peptide	1302
Ligands	6
Water	156
Ramachandran plot	
favored (%)	98
allowed (%)	2
outliers (%)	0
B-factors	31.80
Protein/peptide	31.50
Ligands	18.40
Water	35.60
R.m.s deviations	
Bond lengths (Å)	0.007
Bond angles (º)	1.06

Data collected from a single crystal.
*Highest resolution shell is shown in parenthesis.
